# Cultivation of *Cupriavidus necatorstrains* on hydrolyzed lignocellulosic feedstocks widely available in Europe

**DOI:** 10.1016/j.btre.2025.e00899

**Published:** 2025-05-20

**Authors:** Halima Aliyu Alhafiz, Karin Longus, Rob A.J. Verlinden, Vera Lambauer, Andreas Kruschitz, Regina Kratzer

**Affiliations:** a acib - Austrian Centre of Industrial Biotechnology, Krenngasse 37, 8010 Graz, Austria; b Institute of Biotechnology and Biochemical Engineering, Graz University of Technology, NAWI Graz, Petersgasse 12/I, 8010 Graz, Austria; c Institute of Applied Microbiology, RWTH Aachen University, Worringerweg 1, 52074 Aachen, Germany; d Bioprocess Pilot Facility B.V., Alexander Fleminglaan 1, 2613 AX Delft, the Netherlands

**Keywords:** Lignocellulose saccharification, Adaptive laboratory evolution, *Cupriavidus necator* DSM 545 cultivation, PHA

## Abstract

Today, 85 % of the carbon in organic chemicals and their derivatives comes from fossil sources. Replacing fossil-based materials with sustainable sources requires large quantities of feedstocks and mature technologies. Biorefineries based on lignocellulose have great potential to replace fossil raw materials in the short and medium term. Here we want to pave the way for the bacterium *Cupriavidus necator* as a versatile biotechnological workhorse in future biorefineries. Wheat straw, beech, pine and spruce reflect lignocellulosic biomass from the agricultural waste and wood sectors that is widespread in Europe. Miscanthus was chosen as an emerging energy crop. Lignocellulose feedstocks were pretreated by steam explosion under variable conditions prior to enzymatic hydrolysis. Native *Cupriavidus necator* and a strain adapted by laboratory evolution were shown to grow on 16 filtered lignocellulosic hydrolysates as the sole carbon source and without prior detoxification.

## Highlights

• Wheat straw, beech, pine, spruce and Miscanthus were investigated as feedstocks.
• Physico-chemical pretreatment, enzymatic hydrolysis and solid-liquid separation of biomasses were carried out.
• Obtained fermentable sugars and inhibitory compounds were quantified.
• Growth of *Cupriavidus necator* strains on different lignocellulosic hydrolysates was studied.

## Graphical abstract

**Figure:**
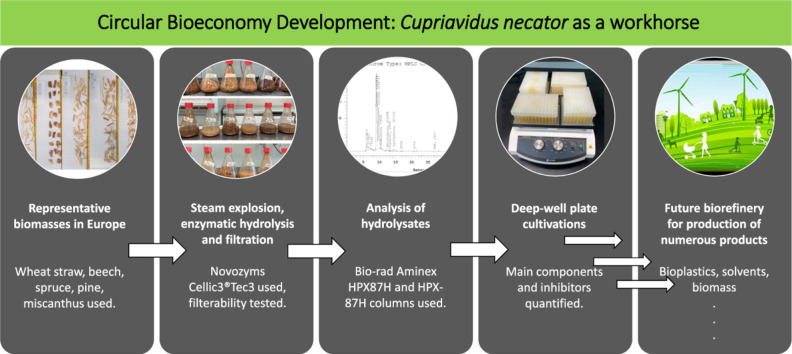

## 1 Introduction

The unsustainable consumption of fossil fuels and fossil-based products depletes resources and releases large quantities of CO_2_, a major greenhouse gas. Efforts to reduce emissions are part of Sustainable Development Goals SDG 7 (Affordable and Clean Energy), SDG 12 (Responsible Consumption and Production), and SDG 13 (Climate Action) in the 2030 Agenda for Sustainable Development (adopted by the UN in 2015). These goals align with the European Green Deal, aiming to make Europe climate neutral by 2050. Achieving this requires sustainable energy and material sources. Global demand for carbon in chemical products is estimated at 450 million tons per year, projected to reach 1000 million tons by 2050. The largest share, 40 %, is used in thermoplastics, with 85 % from fossil sources, 10 % from biomass, and 5 % from recycled materials [1]. Transitioning to a fossil-free future requires renewable carbon sources such as biomass, recycled materials, and CO_2_ captured from industrial processes or the atmosphere. In biorefineries, plant biomasses are processed into bio-based products using conversion technologies. Early biorefineries used food-based feedstocks, but second-generation biorefineries use lignocellulosic biomass that doesn't compete with food production [2]. Modern biorefineries use biomass from agroforestry residues and low-maintenance energy crops grown solely for lignocellulose [3]. Transport routes must be kept short to ensure an economically viable process. We have therefore looked at the availability of lignocellulosic biomass in Europe. The EU-27 processes 1 billion tons of biomass annually, with 50 % from agricultural crops, 27 % from forest biomass, and 9 % from pastures [4]. In 2020, the EU-27 had 180 million hectares of forests, a 6 % increase since 1990 [5]. Annual wood growth is 546 million m³, with potential for an additional 40 to 90 million m³ over the next 10–20 years. However, timber harvesting will require forest restoration and better management due to climate changes [6]. Especially spruce monocultures are vulnerable to pests and climate change and long-term strategies include converting monocultures to mixed forests [7,8]. In 2022, the EU-27 produced 271 million tons of cereals, with wheat (48 %) and maize (19 %) as the main crops [5]. Wheat straw, once used as bedding or burned, is now used for fertilization, energy, and construction. Perennial grasses such as *Miscanthus × giganteus*, which produce high biomass yields, are considered as an alternative [9].

Here, wheat straw, beech, spruce, pine, and Miscanthus were sourced as biomasses available in Europe. Biomasses were hydrolyzed to obtain soluble sugars using well-proven methods such as steam explosion, enzymatic hydrolysis and filtration. The hydrolysates were analyzed by HPLC for sugars and several common inhibitors of microbial growth. The aim was to investigate whether *C. necator* can grow on the different lignocellulosic hydrolysates in principle, or whether the strain is inhibited by components of the hydrolysates.

*C. necator* was chosen as the workhorse for several reasons: (1) Native strains can convert surplus carbon sources, such as sugars and fatty acids into the microbial storage material polyhydroxyalkanoate (PHA, mainly poly-3-hydroxybutyrate PHB), (2) *C. necator* can grow on gas mixtures of H_2_, O_2_ with CO_2_ as sole carbon source and could play a central role in future CO_2_-based biorefineries. (3) *C. necator* strains can be modified using genetic engineering to metabolise non-natural carbon sources such as xylose, mannose or CO [[10], [11], [12], [13]]. (4) The strong carbon storage metabolism of *C. necator* can be redirected towards alternative products like versatile organic solvents [14,15]. The overarching goal is hence to develop *C. necator* as a microbial workhorse in multi-substrate and possibly also multi-product biorefineries.

## 2 Materials and methods

### 2.1 Biomasses, enzymes, chemicals, and strains

Biomasses used as raw materials: milled and dedusted wheat straw (labelled A, broken stalks maximum size 25 × 3 × 2 mm, obtained from Dijkshoorn Bleiswijk B.V., Bleiswijk, The Netherlands); milled *Miscanthus* x *giganteus* (labelled E, broken stalks maximum size 40 × 5 × 2 mm, from Cradle Crops BV, Westdorpe, The Netherlands); beech chips (labelled B, maximum size 20 × 10 × 5 mm, from Sediwood, Domèvre-sur-Durbion and Corbenay, France); pine (labelled D) and spruce chips (labelled D) (maximum size 25 × 7 × 3 mm, from Binderholz, Fügen, Austria) (photos of the biomasses Supplementary material.doc, Figure S1). Additionally, NBSK pulp (from Stora Enso, Finland) was used as raw material. Hydrolytic enzymes Cellic® CTec3 HS (batch VDNI7009) were kindly provided by Novozymes, Lyngby, Denmark and stored at 6 °C. Tryptic soy broth (TSB medium, CASO Bouillon, X938.2) and kanamycin sulfate (≥750 I.U./mg, T832.1) were purchased from Carl Roth (Karlsruhe, Germany). Chemicals for the buffers, HPLC solvents and mineral medium (MM) were from Sigma-Aldrich/Fluka (Vienna, Austria) or Carl Roth and were of the highest purity available. The wild-type *C. necator* H16 DSM 428 (aka ATCC 17,699, NCIB 10,442 aka *Ralstonia eutropha, Alcaligenes eutrophus*) and the constitutive glucose-utilizing strain *C. necator* DSM 545 (aka H1G^+^3, [16]) were from the Leibniz Institute DSMZ and were stored in cryovials containing 0.8 M trehalose at −80 °C [17].

### 2.2 Composition of the raw materials

The content of free sugars, structural carbohydrates, lignin and ash was analyzed before and after the steam explosion. The protocol used was adapted from the National Renewable Energy Laboratory (NREL) and Laboratory analytical procedure (LAP) protocols. The analysis involved grinding of the samples with a conventional kitchen blender (Blokker, The Netherlands) to particle sizes <2 mm. Moisture analysis was performed by infrared moisture balance at 105 °C (VWR, MB160 moisture analyzer, Netherlands) (Supplementary material.doc, Table S1). In addition, acid hydrolysis of the biomass using sugar recovery standards, soluble and insoluble lignin, and organic acids was performed as previously described [18]. The columns used for HPLC analysis were Shodex SP0180 for sugars and Bio-rad Aminex HPX87H for organic acids. Total ash content was determined using an oven LE020K1RN, LE 2/11/R6 (Nabertherm, Germany).

### 2.3 Preparation and pretreatment of the lignocellulose feedstocks

#### 2.3.1 Milling and prewetting of biomasses

Miscanthus and wheat straw were ground by their respective suppliers. Beech, pine, and spruce chips were ground using a VAZ 1100 XL SP shredder (Vecoplan, Birmingham, England) equipped with an 11 mm screen. Biomasses and liquids (water or sulfuric acid) were mixed in a plastic drum (final concentrations of 0.5 % sulfuric acid when applied). The resulting mixtures had moisture contents ranging from 30 % to 60 % (Supplementary material.doc, Table S2,). Prewetted biomasses were stored at 6 °C in plastic bags or plastic buckets prior to steam explosion.

#### 2.3.2 Steam explosion pretreatment

The bench-scale steam explosion was performed using a 40 L pilot steam gun reactor (25 L working volume) supplied by Process- & Industriteknik, Kristianstad, Sweden and installed at BPF (Delft, The Netherlands). The steam gun reactor consisted of a steam generator, a steam inlet valve connected to the steam jacket reactor, a sample inlet valve connected to the steam jacket reactor, a valve to flash off steam from the steam jacket reactor, and a biomass collection cyclone (detailed description in [19]). To preheat the system, two empty sequential experiments were run at process temperature. The resulting condensate was drained to relieve the high pressure in the 4 barg steam lines. The pre-wetted biomass was then added to the steam explosion vessel (typically 20 L of material was used per run) and the air was driven out of the reactor with 4 barg of steam until a temperature of 115 °C was reached. Since steam explosion relies on saturated steam pressure, the efficiency of the pretreatment was increased by degassing the system. Steam explosion was then conducted for 10 min at the saturated steam pressure corresponding to the temperature applied (Table 1). The explosion valve was then opened and the material was transferred to the cyclone by the steam pressure. Finally, the steam-exploded biomass was cooled, weighed and packaged in closed buckets and stored at 6–7 °C. The reactor and cyclone were cleaned with 10 L of water between each run (photos of biomasses after steam explosion in the Supplementary material.doc, Figure S2).

**Table 1:** Steam explosion conditions for used biomasses.

Biomass	Pretreatment Conditions	Sample #
A Wheat straw	No additive, 160 °C	A1
	0.5 % H_2_SO_4_, 160 °C	A2
	No additive, 180 °C	A3
B Beech	0.5 % H_2_SO_4_, 160 °C	B1
	0.5 % H_2_SO_4_, 180 °C	B2
	No additive, 190 °C	B3
C Spruce	0.5 % H_2_SO_4_, 160 °C	C1
	0.5 % H_2_SO_4_, 180 °C	C2
	No additive, 190 °C	C3
D Pine	0.5 % H_2_SO_4_, 160 °C	D1
	0.5 % H_2_SO_4_, 180 °C	D2
	No additive, 190 °C	D3
E Miscanthus	0.5 % H_2_SO_4_, 160 °C	E1
	0.5 % H_2_SO_4_, 180 °C	E2
	No additive, 180 °C	E3
	No additive, 190 °C	E4

### 2.4 Enzymatic hydrolysis

The dry mass content of 1 g sample was analyzed using a moisture analyzer (MA 50, Sartorius AG, Göttingen, Germany) operated at 105 °C. Enzymatic hydrolysis was performed according to the method reported by Novy et al. [20] with modifications. The main difference was that the enzyme cocktail used in the present study was kindly provided by Novozymes (Cellic® CTec3 HS), whereas Novy et al. [20] used an enzyme cocktail produced in-house. Hydrolysis was carried out separately on 16 pretreated samples of wheat straw, beech, spruce, pine, and Miscanthus, as well as on the pulp. Approximately 3 g of Cellic® CTec3 HS were used per 100 g of cellulose, as recommended by the supplier (Novozymes application sheet). Hydrolysis reactions were performed in 500 mL Erlenmeyer flasks with ground stopper. A substrate loading of 15 % by weight (37.5 g dry mass) and 20 mM sodium acetate buffer (pH 4.8, 250 mL total reaction volume) was used. The reaction was initiated by the aseptic addition of 0.44 g (∼0.4 mL) Cellic® CTec3 HS (three times the amount of enzyme was used for the pulp sample). Flasks were incubated at 50 °C and 200 rpm for 72 h (Certomat BS-1). Reactions were stopped in a water bath at 100 °C for 10 min. The residual biomass was separated from the soluble sugar fraction (hydrolysate) by vacuum filtration. The hydrolysates were stored at 4 °C. Experiments were performed in biological triplicates (three hydrolysis reactions were prepared from each pretreated biomass).

### 2.5 Vacuum filtration

The filtration set-up consisted of a Büchner funnel (PP, 390 mL) equipped with two glass fiber filter circles (MN 85/70 BF, retention capacity of 0.6 µm from Macherey-Nagel, purchased from Roth Chemicals, Austria) connected to a 2 L gas wash bottle (type Drechsel, metric fitting 9 mm from DWK Life Sciences DURAN™, purchased from Roth Chemicals) on a balance and further connected to a vacuum pump (type MZ 2C NT 7.0 mbar from Vacuubrand, purchased from Bartelt, Austria). The vacuum was adjusted to 100 mbar with a vacuum gauge (type VAR with manual regulator from Roth Selection, purchased at Roth Chemicals) (set-up in the Supplementary material.doc, Figure S3). The filtration curves (filtrate V_f_ versus time t) were recorded (Supplementary material.exe, excel tabs Filtration curves A1 to Filtration curves pulp; filter cakes, mass balance of filtrations and calculated filter cake resistances are shown in the Figures S4, S5, S6 in the Supplementary material).

### 2.6 HPLC analysis of sugars and metabolites

The hydrolysates were analyzed for sugars (glucose, xylose, arabinose, galactose and mannose and cellobiose), organic acids (formic acid, acetic acid), and sugar degradation products (furfural, hydroxymethylfurfural HMF) by HPLC (Merck-Hitachi LaChrome system with L-7250 autosampler, L-7490 RI detector, L-7400 UV detector). The system was equipped with an Aminex HPX-87H column and an Aminex H guard column. The operation temperature was 65 °C, the flow rate of the mobile phase (5 mM sulfuric acid) was 0.6mL/min. Arabinose and mannose were separated with the same HPLC system but equipped with an Aminex HPX-87P column and an Aminex P guard column (all columns Bio-Rad Laboratories, Hercules, CA, USA). The operating temperature was 80 °C for the main column and room temperature for the guard column. The mobile phase was deionized water at a flow rate of 0.4 mL/min. Experiments were performed in duplicate (HPLC analysis was performed on two hydrolysates per pretreated biomass).

### 2.7 Adaptive laboratory evolution (ALE)

*C. necator* DSM 428 grown in 30 g/L TSB (containing 2.3 g/L glucose) was used as inoculum (initial OD 0.1) for 1 L shaken flasks containing 30 g/L TSB and either 20 g/L glucose or xylose. Weekly, 1 mL of each culture broth was transferred to fresh media. Shaken flask cultures were performed in quadruplicate. As a control, experiments were also performed with TSB medium supplemented with 20 g/L fructose (1 flask per week). After 15 batch cultivations (14 weeks), cells were plated on MM medium agar plates [17] supplemented with 20 g/L glucose or xylose. Single cell colonies were picked and used to inoculate 1 L shaken flasks containing TSB medium supplemented with 20 g/L glucose or xylose (pH of mineral media was 6.8). Cultivation parameters were 30 °C and 110 rpm.

### 2.8 Growth of *C. necator* on lignocellulosic hydrolysates

#### 2.8.1 Deep-well plate cultivation

*C. necator* strains were cultivated in lignocellulosic hydrolysates MM. Mixtures of 5x concentrated MM (200 µL), lignocellulosic hydrolysate (500 µL), preculture (for initial OD_600_ of ∼1.0) were made up with distilled water to final volumes of 1 mL. References was prepared using glucose (5 or 15 g/L) and xylose (5 or 15 g/L) mixtures. All *C. necator* media were supplemented with 50mg/L kanamycin sulfate. Deep-well plate cultivations were performed in 2 mL polypropylene 96 well plates covered with sterile foil (STR-SEAL-PLT polyester 50 µM thickness from SealPLate purchased from Carl Roth). The 96-well plates were shaken at 1300 rpm on a Hei-MIX-Titramax 100 (Heidolph, purchased from Carl Roth). The shaker was placed in a thermostatically controlled room at 30 °C for 3 days.

## 3 Results and discussion

### 3.1 Biomasses selected

Widely available spruce, pine, beech, wheat straw and the emerging energy crop Miscanthus were selected as biomasses in the following experiments (Supplementary material.doc, Figure S1). Biomass contents of cellulose, hemicellulose and lignin vary with growth environment and plant age. Therefore, we determined the composition of the raw biomasses used. The main sugars obtained from the structural carbohydrates cellulose and hemicellulose were ⁠glucose, xylose, galactose, arabinose and mannose (Table 2). Wheat straw showed highest ash (4.3 %), xylose (23.4 %) and arabinose (3.8 %) contents and lowest lignin content (20.1 %), galactose content was 2.2 % and no mannose was found. Wheat straw together with beech and Miscanthus had glucose contents of ∼39 %. Beech biomass had the highest lignin content of 30.0 % and a xylose concentration of 16.8 % (galactose, arabinose and mannose were present in minor concentrations of 2.9, 1.3 and 1.7 %). Spruce and pine had glucose concentrations of 45–46 %, ∼6 % xylose and ∼14 % mannose (and arabinose 2 %). Miscanthus had a relatively high ash content of 2.3 %, a xylose content of 19.6 % (minor contents of galactose, arabinose and mannose of 1.7, 2.3 and 1.2 %). The data were used to evaluate the efficiencies of the subsequent pretreatment and enzymatic hydrolysis steps.

**Table 2:** Composition of biomasses before (in bold) and after pretreatment (conditions see Table 1). (Note that sugar* concentrations refer to the sugars from the structural carbohydrates cellulose and hemicellulose and do not distinguish between cellulose and hemicellulose. Glucose, xylose and arabinose columns show free sugars obtained after pretreatment.).

Sample	Ash (%)	Lignin (%)	⁠Glucose* (%)	Xylose* (%)	Galactose* (%)	Arabinose* (%)	Mannose* (%)	Glucose (%)	Xylose (%)	Arabinose (%)
**A^1^ Wheat straw**	**4.3±0.1**	**20.1±0.2**	**39.2±0.3**	**23.7±0.1**	**2.2±0.1**	**3.8±0.7**	**0**	n.d	n.d	n.d.
A1	4.3 ± 0.0	25.0 ± 0.2	36.8 ± 0.1	23.0 ± 0.2	0	6.0 ± 0.2	0	0.2	0.7	0.1
A2	5.9 ± 0.0	28.9 ± 1.2	40.7 ± 1.0	19.2 ± 0.35	0	0	0	0.1	0.3	0.3
A3	4.6 ± 0.0	28.8 ± 0.5	37.2 ± 0.4	20.7 ± 0.0	0	1.4 ± 0.0	0	0.1	0.4	0.2
**B^2^ Beech**	**1.1±0.2**	**30.0±0.7**	**39.6±0.1**	**16.8±0.1**	**2.9±0.50**	**1.3±0.2**	**1.7±0.1**	n.d	n.d	n.d.
B1	1.2 ± 0.1	38.3 ± 1.5	36.2 ± 0.9	18.1 ± 0.1	0	0	0	0.3	0.9	1.0
B2	1.4 ± 0.2	34.4 ± 0.0	37.8 ± 0.7	7.4 ± 0.4	0	0	0	1.3	11.35	2.8
B3	0.9 ± 0.0	30.3 ± 1.8	35.0 ± 0.0	16.9 ± 0.8	2.2 ± 0.3	1.1 ± 0.02	2.7 ± 0.6	0.2	0.7	0.3
**C^3^ Spruce**	**0.2±0.0**	**26.2±1.6**	**46.1±1.6**	**5.7±0.1**	**2.6±0.1**	**1.8±0.1**	**13.5±0.0**	n.d	n.d	n.d.
C1	0.3 ± 0.0	30.2 ± 0.2	42.6 ± 1.2	2.1 ± 0.1	2.1 ± 0.1	1.0 ± 0.3	13.1 ± 0.7	0.8	4.5	0.7
C2	0.2 ± 0.0	27.0 ± 0.0	49.1 ± 0.7	0.0 ± 1.5	2.5 ± 0.0	0.5 ± 0.2	10.5 ± 0.3	1.8	7.1	0.7
C3	0.3 ± 0.0	27.8 ± 1.2	42.8 ± 1.1	5.2 ± 0.1	2.6 ± 0.1	1.7 ± 0.1	14.9 ± 0.3	0.1	0.4	0.6
**D^4^ Pine**	**0.2±0.1**	**26.5±1.2**	**44.8±0.9**	**5.6±0.2**	**3.0±0.0**	**2.3±0.0**	**14.4±0.3**	n.d	n.d	n.d.
D1	0.3 ± 0.0	35.5 ± 0.3	35.2 ± 0.1	3.6 ± 0.1	2.4 ± 0.2	0.8 ± 0.1	10.5 ± 0.1	1.3	6.5	1.1
D2	0.3 ± 0.0	35.0 ± 1.0	32.4 ± 1.2	3.0 ± 0.1	2.2 ± 0.0	1.1 ± 0.2	10.2 ± 0.2	2.6	10.3	1.0
D3	0.2 ± 0.0	35.8 ± 1.2	39.1 ± 1.0	3.8 ± 0.2	2.1 ± 0.3	1.2 ± 0.0	12.2 ± 0.0	0.1	0.9	0.7
**E^5^ Miscanthus**	**2.5±0.0**	**26.2±0.7**	**39.2±0.5**	**19.6±0.1**	**1.7±0.2**	**2.3±0.0**	**1.2±0.0**	n.d	n.d	n.d.
E1	3.2 ± 0.1	25.8 ± 0.2	39.1 ± 0.1	17.4 ± 0.4	3.1 ± 0.3	1.9 ± 0.4	0	0.4	2.4	0.7
E2	3.0 ± 0.0	26.0 ± 0.3	39.2 ± 0.1	16.1 ± 0.1	2.9 ± 0.0	1.2 ± 0.1	0	1.1	3.8	0.9
E3	2.9 ± 0.0	26.0 ± 0.1	39.1 ± 0.3	19.2 ± 0.5	3.2 ± 0.1	2.0 ± 0.0	0	0.1	0.3	0.5
E4	3.1 ± 0.2	26.8 ± 0.2	38.3 ± 0.2	19.9 ± 0.0	2.2 ± 0.3	1.4 ± 0.0	0	0.1	0.9	0.5
pulp	0.2 ± 0.0	1.6 ± 0.2	75.9 ± 0.2	7.5 ± 0.2	0	4.1 ± 0.0	8.5 ± 0.0	0	0	0

^1^Deviation from e.g. [33] <1 %. ^2^Deviation from e.g. [32] <3 %.

^3^Deviation from e.g. [34] <3 %. ^4^Deviation from e.g. [35] <3 %.

^5^Relatively low glucose* concentration indicated young plants [36]. n.d. not determined.

### 3.2 Pretreatment of biomasses

Biomass steam explosion uses high temperature steam and pressure differences to loosen the structure of lignocellulosic biomass, leading to increased accessibility of cellulose and hemicellulose for the subsequent enzymatic hydrolysis. We compared steam explosion pretreatment without acid at higher temperatures and at lower temperatures with 0.5 % H_2_SO_4_ (conditions summarized in Table 1). In general, a decrease in hemicellulose and lignin is expected as a result of the pretreatment (photos of biomasses after steam explosion Supplementary material.doc, Figures S2). Table 2 shows the composition of the biomasses before and after pretreatment (only main types of monosaccharides are shown). The partial degradation of hemicellulose is generally seen as a decrease in the xylose* fraction (xylose found in hemicellulose) and an increase in free xylose. This effect was particularly pronounced for wood samples B2 (beech chips steam exploded at elevated temperature of 180 °C, with H_2_SO_4_), C1, C2, D1 and D2 (spruce and pine steam exploded with H_2_SO_4_). For most of the samples, the lignin content (% of total biomass) appeared to increase. However, comparisons of biomass composition before and after the pretreatment step are difficult to interpret due to washing losses of solubles during steam explosion and a possible formation of pseudolignin by rearrangement and polymerization of furfural and HMF [21]. The pretreated biomass samples were subjected to enzymatic hydrolysis.

### 3.3 Enzymatic hydrolysis

Enzymatic hydrolysis of pretreated biomass samples (enzyme cocktail Cellic® CTec3 HS from Novozymes) was used to release soluble sugars. The slurry was subjected to filtration and the supernatant was analyzed for monosaccharides and inhibitory compounds. The monosaccharides, the sugar degradation products HMF and furfural and the organic acids formic and acetic acid in the filtrates were analyzed. Wheat straw hydrolysates A2 and A3 had the highest glucose concentrations of 30 and 23 g/L, equal to hydrolysis yields of 57 and 39 % (Table 3), respectively. This was followed by the Miscanthus hydrolysates E2 and E4 with glucose concentrations of 14 and 15 g/L (hydrolysis yields of 36 and 34 %). The hydrolyzed wood biomasses generally yielded lower glucose concentrations between 4 and 7 g/L, with the exception of the hydrolysates of beech biomass B3, which yielded 10 g/L glucose (hydrolysis yields below 30 %). Incomplete hydrolysis was indicated by the low sugar concentrations, but was also evident in the filter cakes obtained (Figure S4 in the Supplementary material). The low enzymatic hydrolysis yields can be partly explained by the relative high loading of 15 % dry biomass in the hydrolysis reaction slurry. Loadings of 15 % of dry weight had previously led to maximum hydrolysis yields of 60 to 82 % for different biomasses [22]. Too large chip sizes (beech 20 × 10 × 5 mm, pine and spruce 25 × 7 × 3 mm) and stem sizes (wheat straw 25 × 3 mm and Miscanthus 40 × 5 mm) further contributed to incomplete hydrolysis. Measures to increase hydrolysis yields are lower solid loads and size reduction of the starting materials. In the present study, the enzyme loading was according to the supplier (Novozyme application sheet). In general, the hydrolysis yield can be improved by increasing the enzyme loading and the mixing quality. Novy et al. [20] reported that increasing the enzyme concentration by 20 % led to a 7 % increase in the hydrolysis yield.

**Table 3:** Sugar monomers, organic acids, furfural and HMF in the filtrates after enzymatic hydrolysis. The hydrolysis yields were calculated from the biomasses obtained after steam explosion (Table 2).

Sample	Glucose (g/L)	Xylose (g/L)	Arabinose (g/L)	Mannose (g/L)	Formic acid (g/L)	Acetic acid (g/L)	Furfural (g/L)	HMF (g/L)	Hydrolysis yield (%)
Wheat straw A1	12.5 ± 0.4	5.1 ± 0.2	1.00 ± 0.02	0.13 ± 0.01	1.35 ± 0.02	0.10 ± 0.01	0.25 ± 0.23	0.11 ± 0.02	19
A2	30.2 ± 1.8	19.8 ± 1.0	0.47 ± 0.01	1.45 ± 0.15	1.68 ± 0.07	0.20 ± 0.01	1.07 ± 0.09	0.36 ± 0.02	57
A3	22.9 ± 0.2	12.0 ± 0.2	0.50 ± 0.02	0.44 ± 0.34	1.09 ± 0.1	0.11 ± 0.00	0.03 ± 0.00	0.05 ± 0.01	39
Beech B1	6.0 ± 0.4	6.4 ± 0.5	0.32 ± 0.02	0.10 ± 0.04	0.12 ± 0.01	0.13 ± 0.01	0.57 ± 0.51	0.18 ± 0.05	16
B2	6.6 ± 0.2	14.1 ± 0.1	0.34 ± 0.01	0.11 ± 0.01	0.31 ± 0.00	0.22 ± 0.00	1.19 ± 0.25	1.04 ± 0.80	29
B3	10.3 ± 0.1	13.3 ± 0.0	0.08 ± 0.00	0.06 ± 0.00	0.34 ± 0.01	0.23 ± 0.00	0.84 ± 0.29	0.23 ± 0.01	27
Spruce C1	4.1 ± 0.0	9.3 ± 0.1	1.28 ± 0.02	5.82 ± 0.11	0.03 ± 0.00	0.09 ± 0.00	0.34 ± 0.04	0.41 ± 0.02	23
C2	5.1 ± 0.1	11.4 ± 0.6	0.79 ± 0.04	6.95 ± 0.70	0.00 ± 0.00	0.11 ± 0.00	1.02 ± 0.00	1.47 ± 0.07	28
C3	5.4 ± 0.1	3.4 ± 0.2	1.09 ± 0.15	1.38 ± 0.12	0.39 ± 0.24	0.04 ± 0.03	0.49 ± 0.27	1.04 ± 0.88	12
Pine D1	3.8 ± 0.1	8.8 ± 0.2	1.40 ± 0.03	3.75 ± 1.84	0.02 ± 0.00	0.09 ± 0.00	0.21 ± 0.01	0.30 ± 0.01	23
D2	4.8 ± 0.1	14.7 ± 1.7	0.97 ± 0.11	8.40 ± 0.78	0.07 ± 0.07	0.09 ± 0.03	0.46 ± 0.20	0.93 ± 0.76	41
D3	4.7 ± 0.6	3.4 ± 0.3	0.73 ± 0.06	3.44 ± 1.89	0.15 ± 0.01	0.43 ± 0.03	0.43 ± 0.03	0.26 ± 0.01	15
Miscanthus E1	10.0 ± 0.8	7.9 ± 0.9	1.01 ± 0.11	0.00 ± 0.00	0.23 ± 0.02	0.61 ± 0.04	0.61 ± 0.04	0.14 ± 0.00	21
E2	13.8 ± 0.1	12.6 ± 0.1	0.77 ± 0.01	3.60 ± 0.86	0.35 ± 0.00	2.17 ± 0.52	2.17 ± 0.52	0.57 ± 0.00	36
E3	12.4 ± 0.0	10.8 ± 0.1	0.86 ± 0.01	0.92 ± 0.66	0.43 ± 0.00	0.33 ± 0.02	0.33 ± 0.02	0.12 ± 0.01	26
E4	15.1 ± 0.5	14.2 ± 0.2	0.35 ± 0.01	1.30 ± 0.39	0.52 ± 0.00	1.43 ± 0.30	1.43 ± 0.30	0.25 ± 0.01	34
Pulp	62.0 ± 2.0	10.7 ± 0.3	0.54 ± 0.07	2.78 ± 0.11	0.01 ± 0.00	0.00 ± 0.00	0.00 ± 0.00	0.00 ± 0.00	52

Low sugar concentrations in the wood hydrolysates are not only due to low enzyme yields, but also reflect the sugar degradation during steam explosion and the loss of soluble components in the aqueous fraction after steam explosion. The inhibitors formed during pretreatment by steam explosion were analyzed after enzymatic hydrolysis to quantify the amounts of potentially inhibitory compounds in the subsequent cultivation trials (Table 3). Wheat straw in particular showed relatively high formic acid concentrations of 1.1 to 1.7 g/L. As expected, the concentrations of furfural and HMF were highest in the heavily pretreated (acid and higher temperature) biomass samples. The highest concentrations of 2.2 g/L furfural and 1.5 g/L HMF were found in the samples of Miscanthus (E2) and spruce (C2), respectively.

Overall, the hollow stems of the cereal grass wheat straw proved to be the most useful lignocellulosic raw material in terms of sugar yield. The highest glucose yields were obtained with wheat straw sample A2 (pre-treated with acid at elevated temperatures). The woody biomasses generally had low glucose yields compared to the grasses. All hydrolysates were used as carbon sources for the cultivation of *C. necator* strains.

### 3.4 Growth of *C. necator* strains on lignocellulosic hydrolysates

#### 3.4.1 Ability of C. necator to grow on carbohydrates

**Fig. 1:**
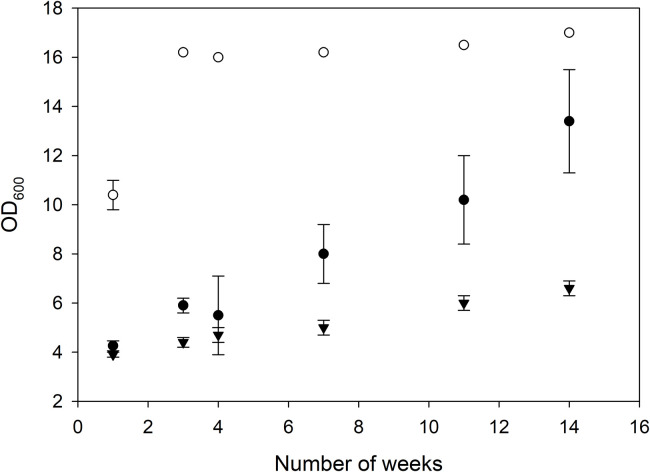
Growth of *C. necator* H16 in serial shake flask cultivations in TSB containing either additional 20 g/L glucose (full circles), 20 g/L xylose (full triangles) or 20 g/L fructose (open circles).

*C. necator* H16 has a limited carbohydrate utilization range and cannot assimilate glucose or xylose, key sugars in non-edible cellulose. A glucose-utilizing mutant, *C. necator* DSM 545 (H1G^+^3), was created from *C. necator* H16 by UV mutagenesis [16]. Previous studies showed fast adaptability of *C. necator* H16 to glucose utilization (e.g., [23,24]). In this study, *C. necator* H1G^+^3 and *C. necator* H16 were tested on lignocellulose hydrolysates. First, *C. necator* H16 was subjected to adaptive laboratory evolution (ALE) for glucose and xylose utilization in serial batch cultivations. The adaptation to glucose was reflected in a steady increase in the OD_600_ (Fig. 1). Growth on xylose increased 1.5-fold in TSB after 14 re-cultivations, but no growth occurred in MM. Increased growth in cultivations with xylose (and fructose) was most probably due to adaptation to the glucose-content in the TSB medium.

#### 3.4.2 Ability of C. necator to grow on lignocellulosic hydrolysates

**Fig. 2:**
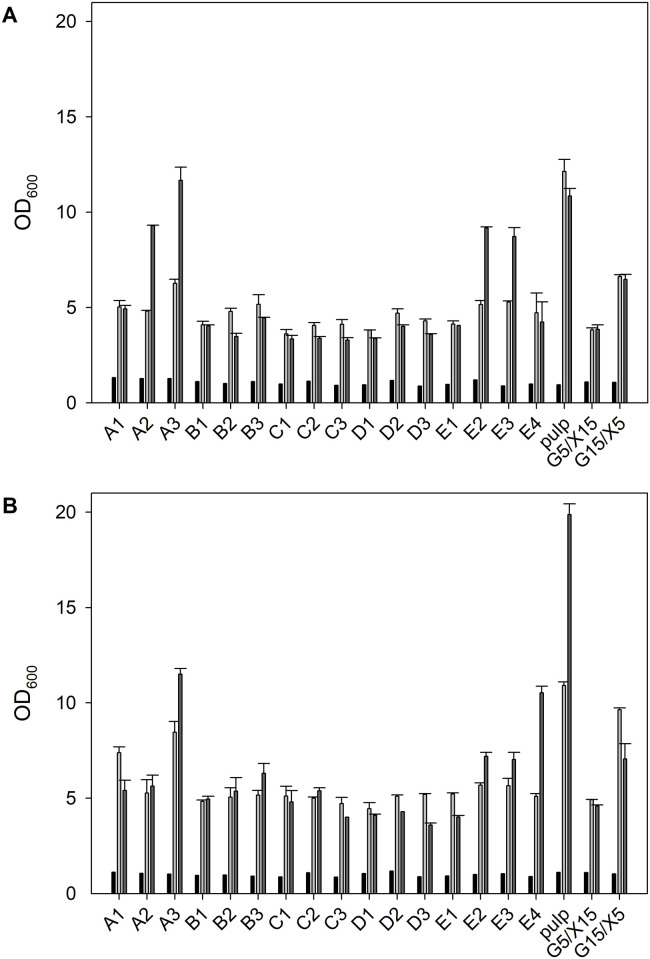
Growth of *C. necator* strains (OD_600_) on different hydrolysates, black bar at 0 h (inoculum), light gray bar at after 1 day (26 h), dark gray bar after 2 days of cultivation (47 h). For comparison, growth in a medium containing 2.5 g/L glucose and 7.5 g/L xylose (G2.5/X7.5) and 7.5 g/L glucose and 2.5 g/L xylose (G7.5/X2.5). (A) *C. necator* H16 (DSM 428) adapted to glucose by adaptive laboratory evolution. (B) *C. necator* H1G^+^3 (DSM 545).

**Fig. 3:**
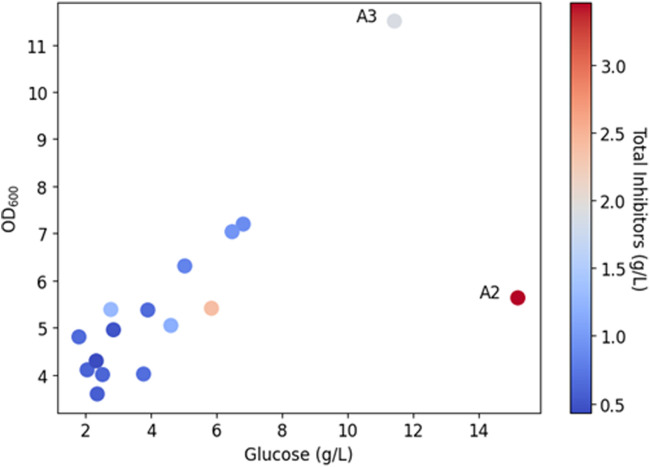
The growth of *C. necator* H1G^+^3 (DSM 545) (OD_600_) on different hydrolysates correlates with the corresponding glucose concentration. Each dot represents one deep-well plate cultivation (in biological duplicates). Dot color represents the total inhibitor concentration (blue = low, red = high; total inhibitor concentration reflects the sum of acetic acid, formic acid, furfural, HMF).

Numerous by-products of lignocellulose pretreatment and hydrolysis are known inhibitors of microbial growth. Growth-inhibition naturally depends on inhibitor concentrations and type of microorganism. In Table 3, the concentrations of formic acid, acetic acid, furfural and HMF, known as inhibitors to microbial growth, are listed [25]. Lignin degradation products such as aromatic alcohols, aldehydes and acids were not quantified. We have tested all hydrolysates diluted to 50 % in deep-well plate cultivations with *C. necator* strains (H1G^+^3 and to glucose adapted H16) (Fig. 2). In all deep-well plate cultivations the optical density increased at least 5-fold after one day. Growth correlated with the glucose concentration (Fig. 3). The most obvious difference between the strains were higher OD_600_ values obtained for the *C. necator* H1G^+^3 in cultivations with high glucose concentrations (pulp sample, reference sample with 7.5 g/L glucose and 2.5 g/L xylose (G7.5/X2.5)). This indicated that the random mutant *C. necator* H1G^+^3 still grew faster on glucose than the adapted *C. necator* H16 strain. No major correlations to the concentrations of the inhibitors formic acid, acetic acid, furfural and HMF were found (tested as multivariate analysis of OD_600_ values versus glucose and different inhibitors, not shown). However, comparing the growth (Fig. 2) in hydrolysates A2 and A3, it is seen that the cells grew to a higher OD in A3 compared to A2 despite the fact that the glucose concentration was higher in A2. Examining the different inhibitor concentrations of A2 it is seen that the sum of the analyzed inhibitors is the highest of all samples in A2 (Fig. 3) leading to approximately 50 % reduction in the expected OD_600_ (in comparison to hydrolysate A3).

Furthermore, all hydrolysates were diluted to a glucose concentration of ∼2 g/L in the culture medium. Very similar OD_600_ values were obtained with all hydrolysates and reference samples (Supplementary material, Figure S7). Under the tested conditions (hydrolysates in Table 3 diluted to 50 % and to 2 g/L glucose) no major inhibition was experienced. The highest furfural and HMF concentrations in the culture broths were of 1.1 g/L (Table 3, E2 diluted to 50 %) and 0.74 g/L (Table 3, C2 diluted to 50 %), respectively (Fig. 2). These concentrations have previously shown to completely prevent growth of the alternative PHA producer *Paraburkholderia sacchari* IPT 101 [26].

## 4 Conclusions

The soil bacterium *C. necator* is a promising workhorse for future biorefineries. It grows heterotrophically and lithotrophically, and it can convert excess carbon sources into polyhydroxyalkanoate (mainly poly-3-hydroxybutyrate, PHB), a microbial bioplastic that makes up 80–90 % of the total microbial biomass [27]. Although the alternative PHB producer *Paraburkholderia sacchari* seems more practical due to its ability to metabolize a broader range of sugars, our results in comparison to literature data [26] indicate a lower inhibitor tolerance of *P. sacchari* compared to *C. necator*. Furthermore, the availability of genetic tools (e.g. [28]) for *C. necator* enables an expansion of the substrate spectrum for e.g. xylose or mannose [[11], [12], [13]] or the product spectrum [14,15].

A critical factor in bioprocessing is the product concentration in the fermentation broth. The highest reported cell density of 281 g/L with a PHB content of 232 g/L was obtained using glucose as the carbon source in a fed-batch fermentation [29]. To achieve such high cell concentrations, high sugar concentrations are required. Despite optimized hydrolysis protocols (here biomass size reduction, increased enzyme loading), these sugar concentrations are generally not obtainable from hydrolysed lignocellulose without prior concentration [30]. Concentration of the sugar solution would, however, also lead to higher inhibitor concentrations and thus the need for an inhibitor removal step. To supply cell densities of >200 g/L cell dry weight with sufficient oxygen in large scale bioreactors, high mass transfer and thus high mixing energy is required. These questions can only be answered by a detailed life cycle analysis of the process. In any scenario, a biorefinery based on *C. necator* requires a genetically modified strain that utilizes xylose (and possibly also mannose), most probably the concentration and detoxification of the sugar solution and, in case of PHA as product, a sustainable solution for PHA isolation [31].

## Declaration of generative AI and AI-assisted technologies in the writing process

During the preparation of this work the author(s) used DeepL.com for grammar checking. After using this tool/service, the author(s) reviewed and edited the content as needed and take(s) full responsibility for the content of the publication.

## Funding

This project has received funding from the European Union’s Horizon 2020 Research and Innovation programme under grant agreements No. 953206 (Bionanopolys) and under the Marie Skłodowska Curie grant agreement No. 955740 (ConCO2rde). The COMET center ACIB: Next Generation Bioproduction was funded by BMK, BMDW, SFG, Standortagentur Tirol, Government of Lower Austria und Vienna Business Agency in the framework of COMET-Competence Centers for Excellent Technologies. The COMET-Funding Program was managed by the Austrian Research Promotion Agency FFG (No. 872161).

## Author agreement

All authors (Halima Aliyu Alhafiz, Karin Longus, Rob Verlinden, Vera Lambauer, Andreas Kruschitz, Regina Kratzer) agree to the final version of this manuscript and to the submission to Biotechnology reports.

## CRediT authorship contribution statement

**Halima Aliyu Alhafiz:** Writing – original draft, Visualization, Validation, Investigation, Formal analysis, Data curation. **Karin Longus:** Validation, Methodology, Investigation, Data curation. **Rob A.J. Verlinden:** Writing – review & editing, Resources, Project administration, Methodology, Investigation, Funding acquisition, Data curation, Conceptualization. **Vera Lambauer:** Validation, Methodology, Investigation, Data curation. **Andreas Kruschitz:** Writing – review & editing, Methodology, Investigation, Data curation. **Regina Kratzer:** Writing – review & editing, Writing – original draft, Visualization, Supervision, Resources, Project administration, Funding acquisition, Conceptualization.

## Declaration of competing interest

The authors declare that they have no known competing financial interests or personal relationships that could have appeared to influence the work reported in this paper.

## Data Availability

Data is found in the Supplementary files to this article.

## Appendix Supplementary materials

Image, application 1

Image, application 2
