# Supplementary material for: Cultivation of Cupriavidus necatorstrains on hydrolyzed lignocellulosic feedstocks widely available in Europe
Source: Biotechnol Rep (Amst). 2025 May 20;47:e00899. doi: 10.1016/j.btre.2025.e00899 (PMC12178924; doi:10.1016/j.btre.2025.e00899)
Supplement: Supplementary file 2 [file mmc2.docx]

**Supplementary material**

**Table of contents**

**1. Characterization of the raw biomasses**

*Figure S1.* Photos of chopped raw wheat straw, beech, spruce, pine, miscanthus biomasses with scale.

*Table S1.* Moisture content (%) of the raw biomass samples.

**2. Characterization of the pretreated biomasses**

*Table S2*. Moisture content (%) of biomass samples after pretreatment.

*Figure S2A*. Photos of pretreated wheat straw with scale.

*Figure S2B*. Photos of pretreated beech with scale.

*Figure S2C.* Photos of pretreated spruce with scale.

*Figure S2D*. Photos of pretreated pine with scale.

*Figure S2E*. Photos of pretreated miscanthus with scale.

**3. Hydrolysis and filtration**

*Figure S3.* Vacuumfiltration set-up.

*Figure S4*. Photos of filter cakes obtained by lab-scale vacuum filtration.

*Figure S5*. Filtration mass balances including filtrate, dried filter cake and residual filtrate in the wet filter cake.

*Figure S6*. Correlation of OD600 to glucose concentration in lignocellulose hydrolysates (including pulp sample). Mineral media with 50% lignocellulosic hydrolysates in deep-well plate cultivations of *C. necator* H1G^+^3 (full circles) and *C. neactor* H16 adapted to glucose (open circles).

**1. Characterization of the raw biomasses**


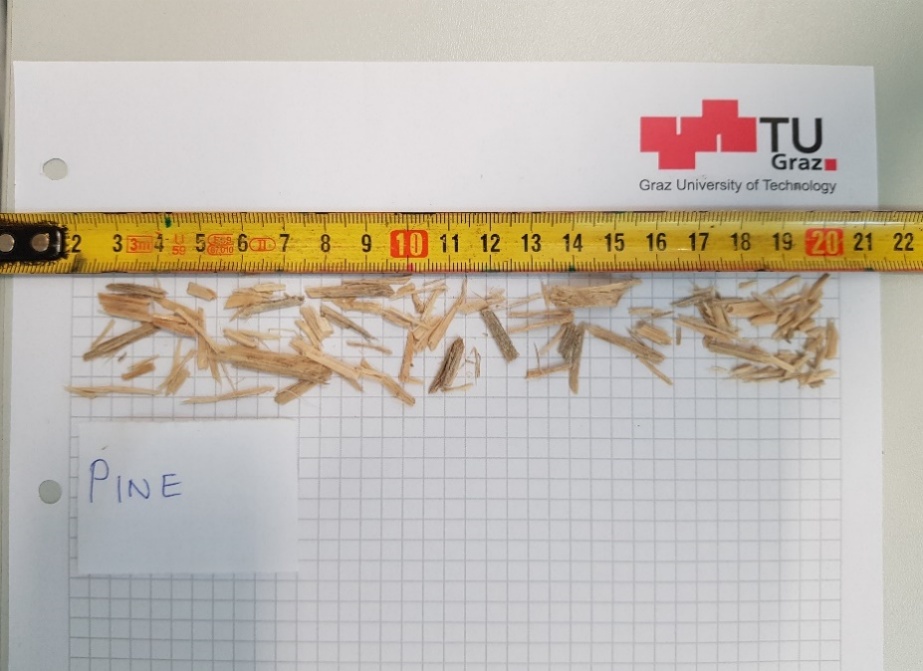

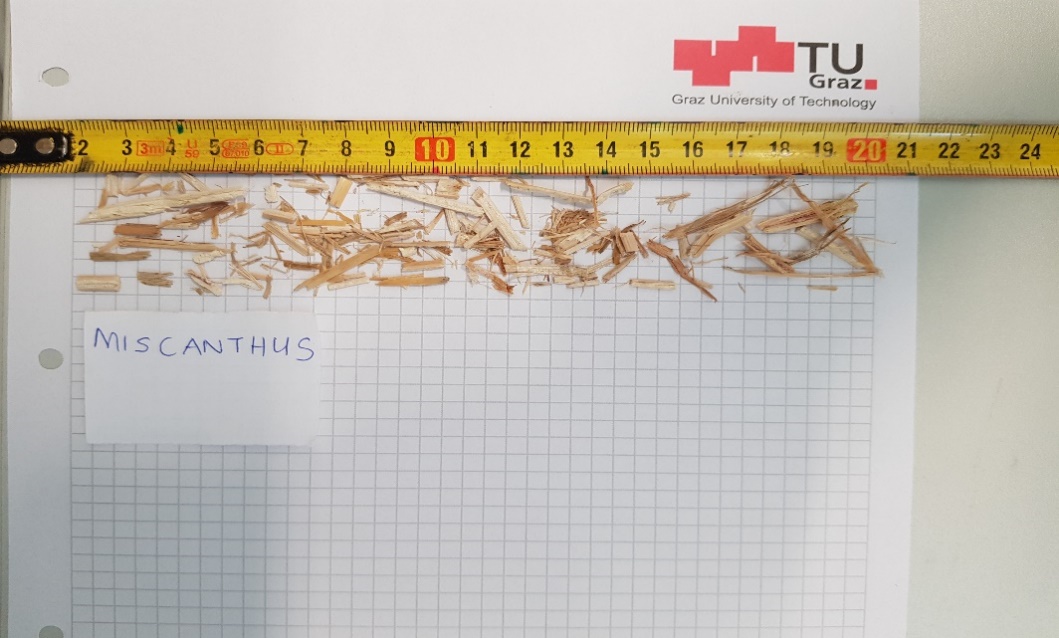

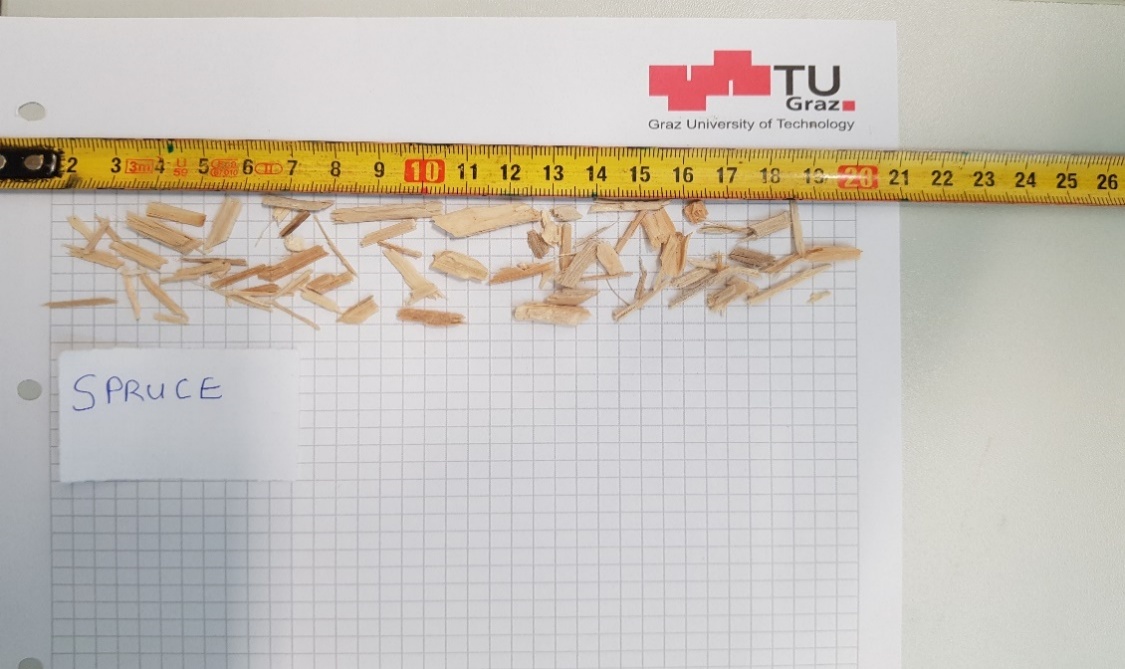

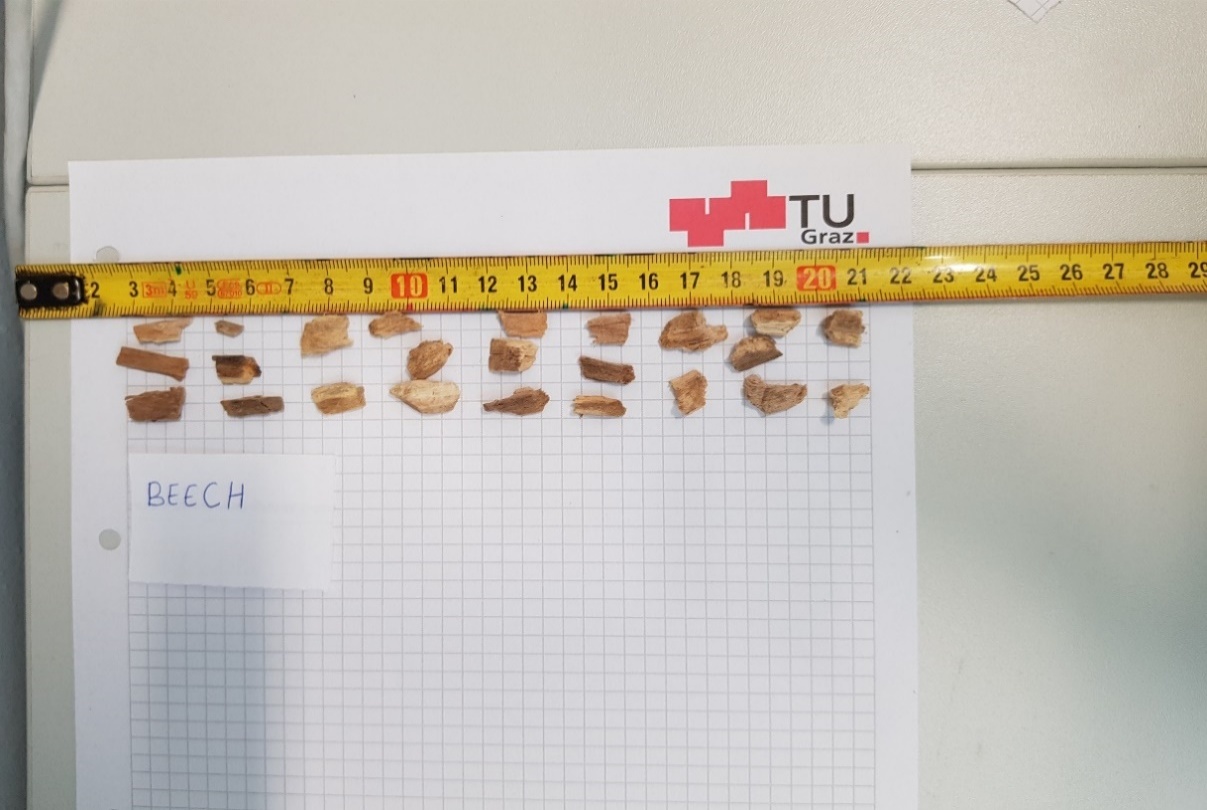

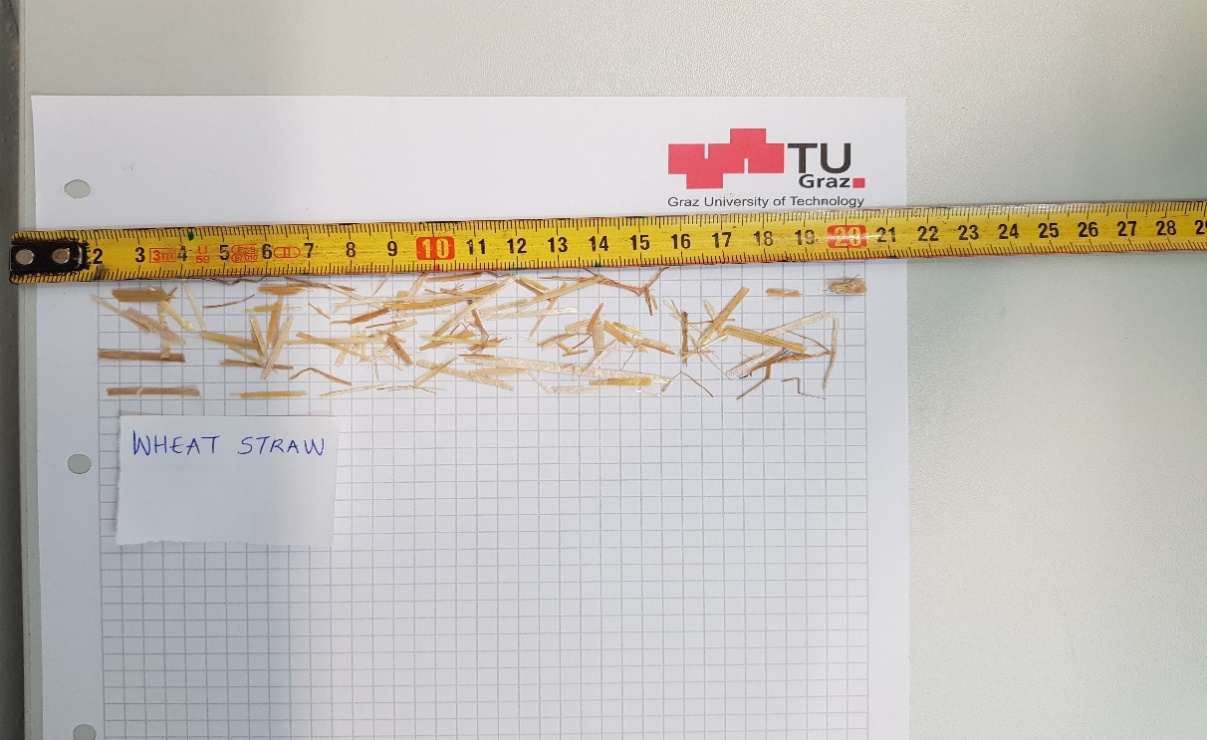


*Figure S1*. Photos of chopped raw wheat straw, beech, spruce, pine, miscanthus biomasses with scale.

*Table S1.* Moisture content (%) of the raw biomass samples.

| **Sample** | **Biomass** | **Moisture content (%)** |
| --- | --- | --- |
| A | Wheat straw | 6.6 |
| B | Beech | 30.4 |
| C | Spruce | 29.1 |
| D | Pine | 38.4 |
| E | Miscanthus | 11.2 |
|  | NBKS pulp | 3.0 |

**2. Characterization of the pretreated biomasses**

Table S2. Steam explosion conditions for used biomasses.

| Biomass | Pretreatment Conditions | Sample # | Moisture content (%) |
| --- | --- | --- | --- |
| A Wheat straw | No additive, 160°C | A1 | 58.0 |
|  | 0.5% H_2_SO_4_, 160°C | A2 | 41.3 |
|  | No additive, 180°C | A3 | 46.4 |
| B Beech | 0.5% H_2_SO_4_, 160°C | B1 | 45.9 |
|  | 0.5% H_2_SO_4_, 180°C | B2 | 51.9 |
|  | No additive, 190°C | B3 | 48.4 |
| C Spruce | 0.5% H_2_SO_4_, 160°C | C1 | 44.5 |
|  | 0.5% H_2_SO_4_, 180°C | C2 | 44.2 |
|  | No additive, 190°C | C3 | 41.3 |
| D Pine | 0.5% H_2_SO_4_, 160°C | D1 | 49.9 |
|  | 0.5% H_2_SO_4_, 180°C | D2 | 51.0 |
|  | No additive, 190°C | D3 | 51.7 |
| E Miscanthus | 0.5% H_2_SO_4_, 160°C | E1 | 38.1 |
|  | 0.5% H_2_SO_4_, 180°C | E2 | 45.2 |
|  | No additive, 180°C | E3 | 43.5 |
|  | No additive, 190°C | E4 | 48.3 |


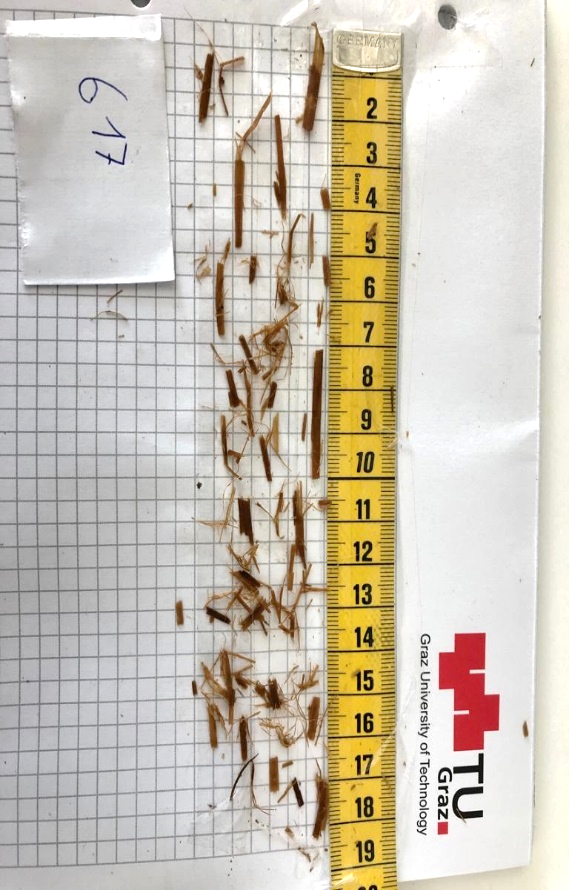

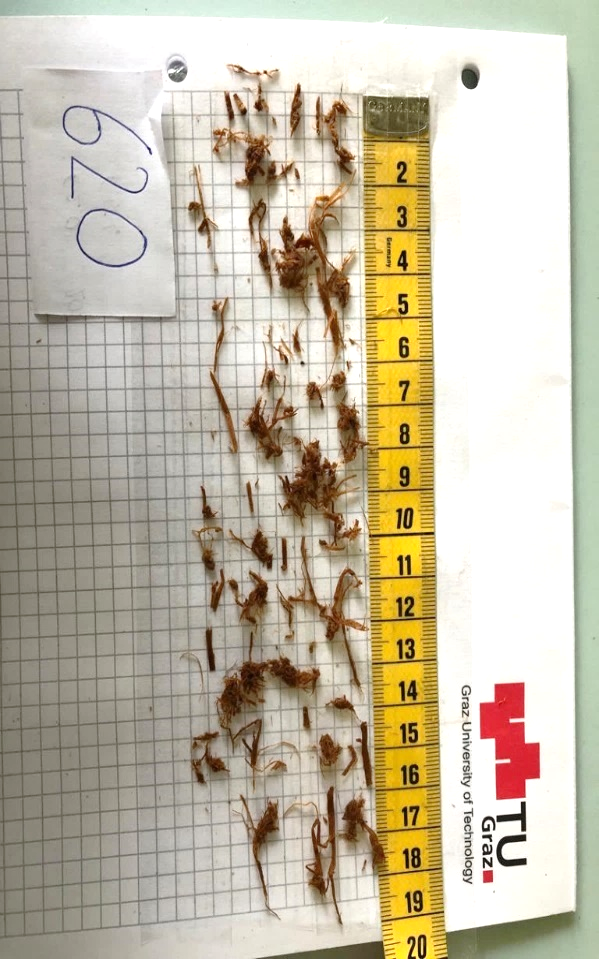

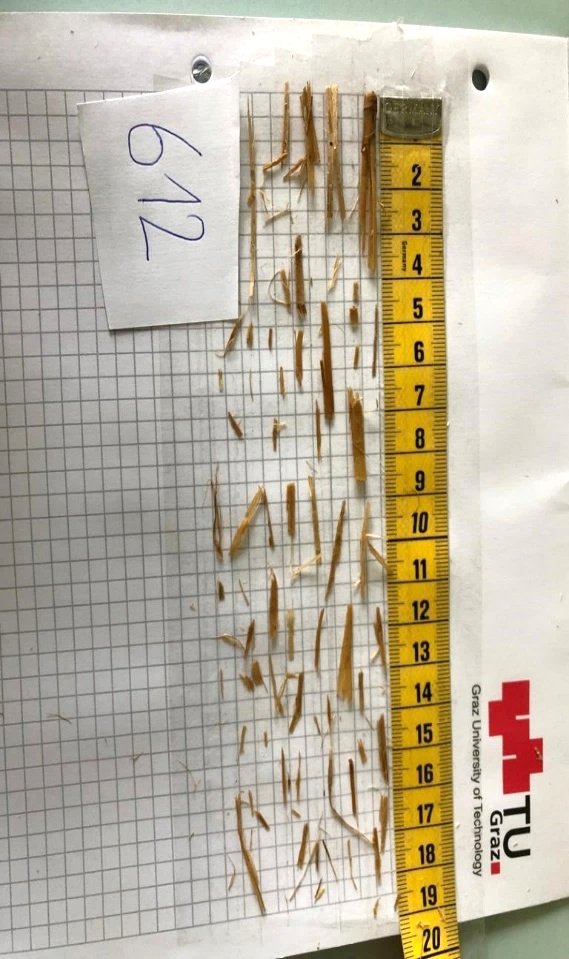


*Figure S2A*. Photos of pretreated wheat straw with scale. 612/A1 steam explosion (180°C/10 minutes); 620/A2 0.5% H_2_SO_4_ + steam explosion (160°C/10 minutes); 617/A3 steam explosion (160°C/10 minutes).


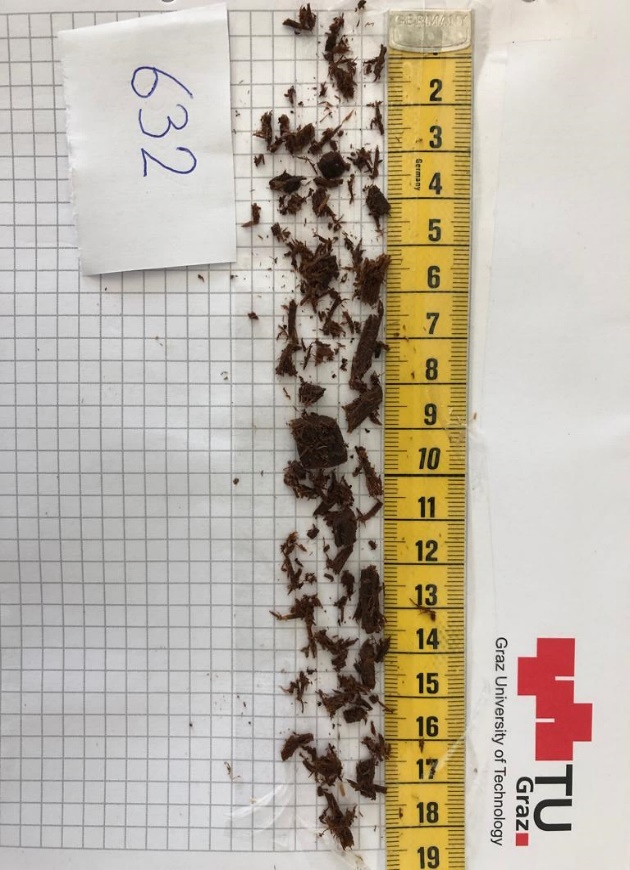

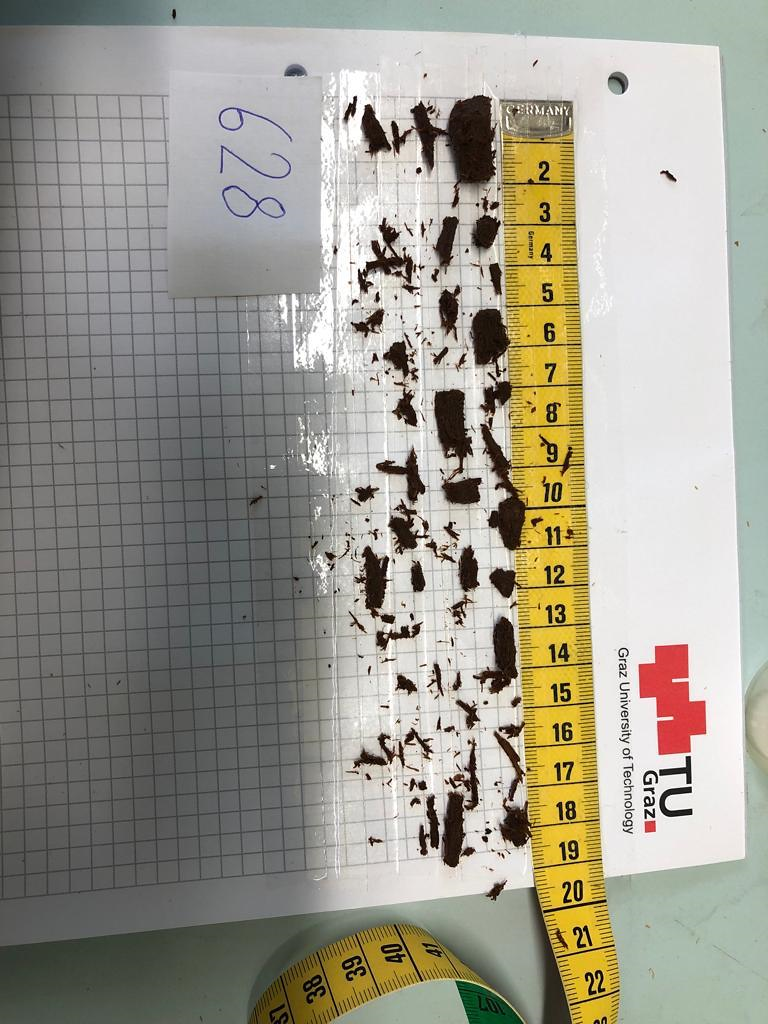

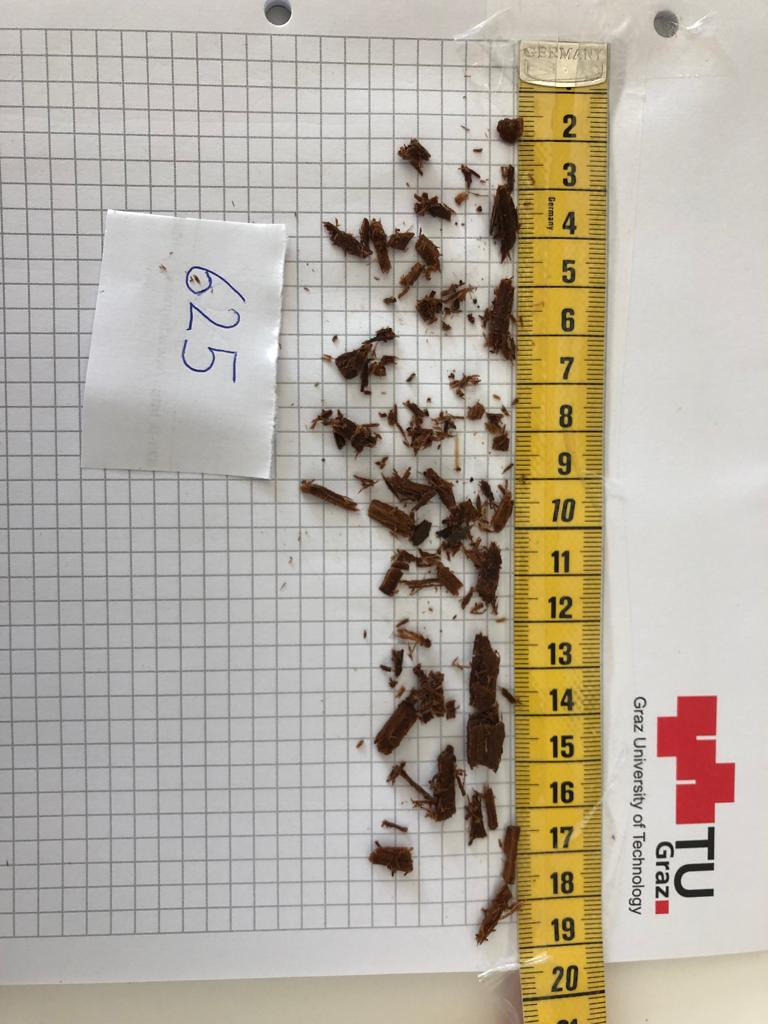


*Figure S2B*. Photos of pretreated beech with scale. 625/B1 0.5% H_2_SO_4_ + steam explosion (160°C/10 minutes); 628/B2 0.5% H_2_SO_4_ + steam explosion (180°C/10 minutes); 632/B3 steam explosion (190°C/10 minutes).


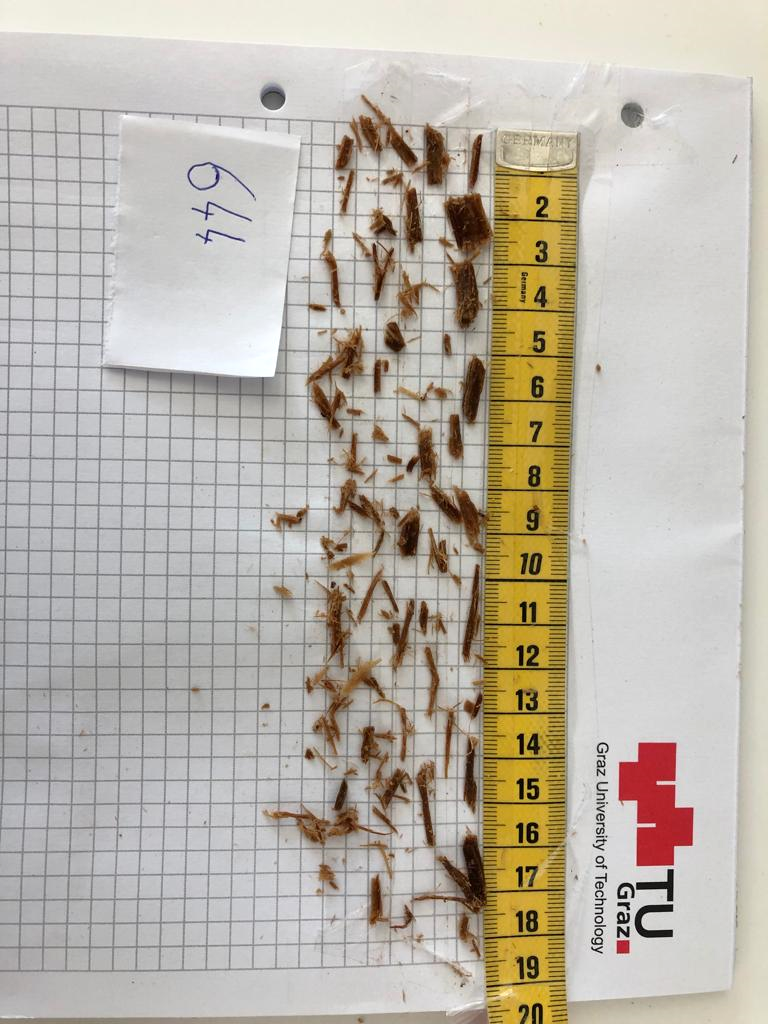

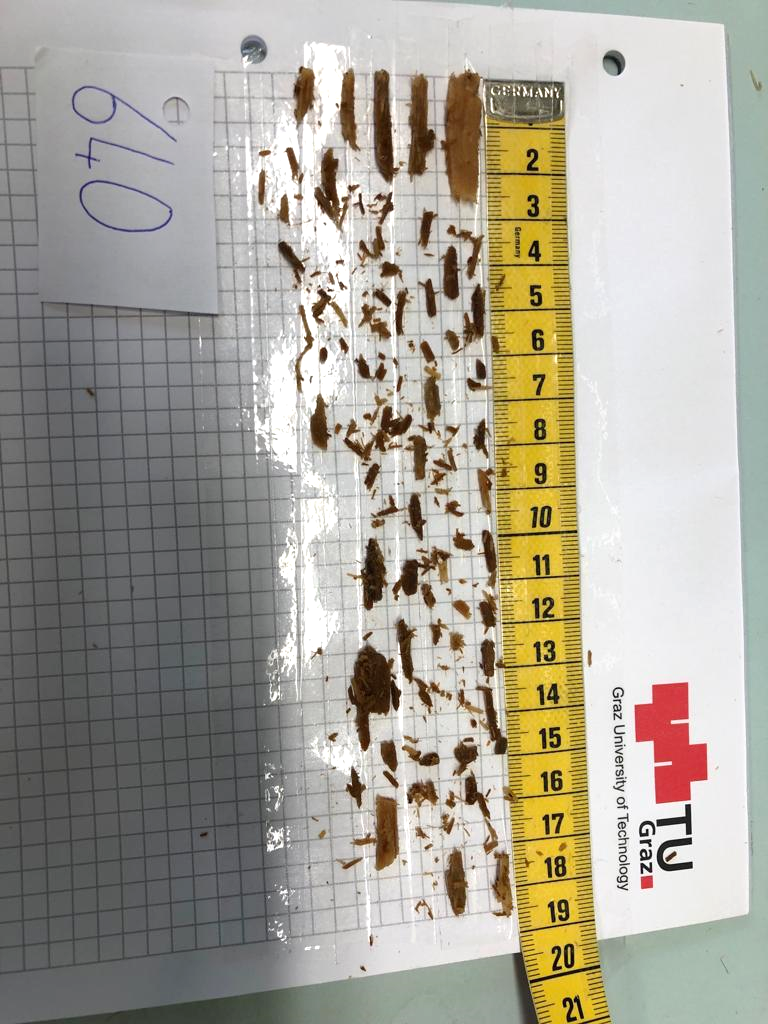

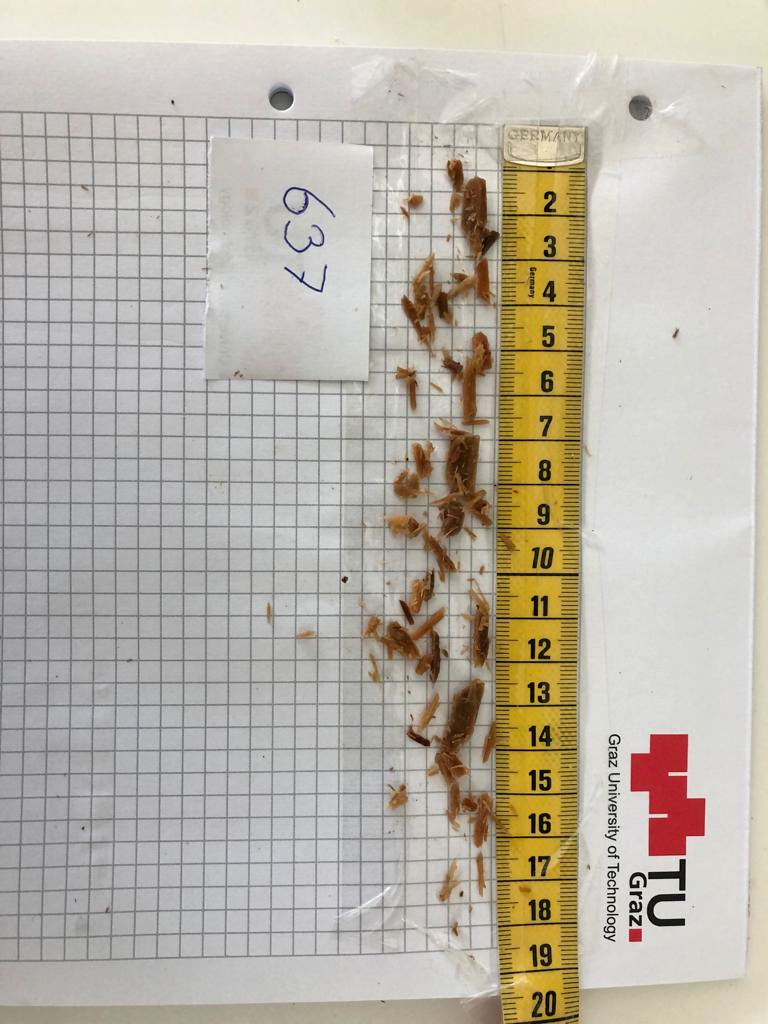


*Figure S2C*. Photos of pretreated spruce with scale. 637/C1 0.5% H_2_SO_4_ + steam explosion (160°C/10 minutes); 640/C2 steam explosion (190°C/10 minutes); 644/C3 0.5% H_2_SO_4_ + steam explosion (180°C/10 minutes).


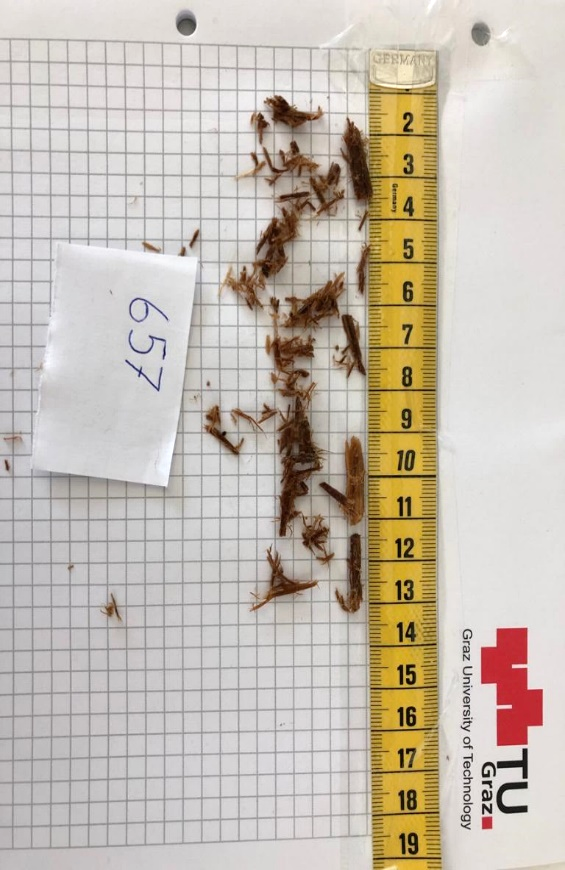

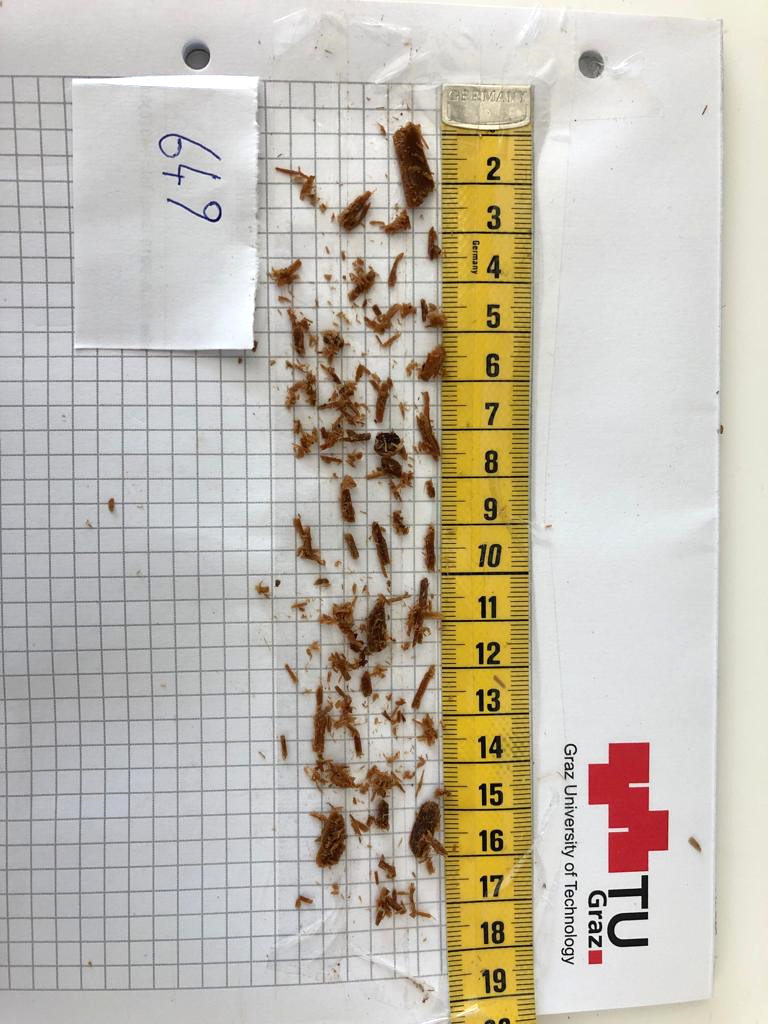

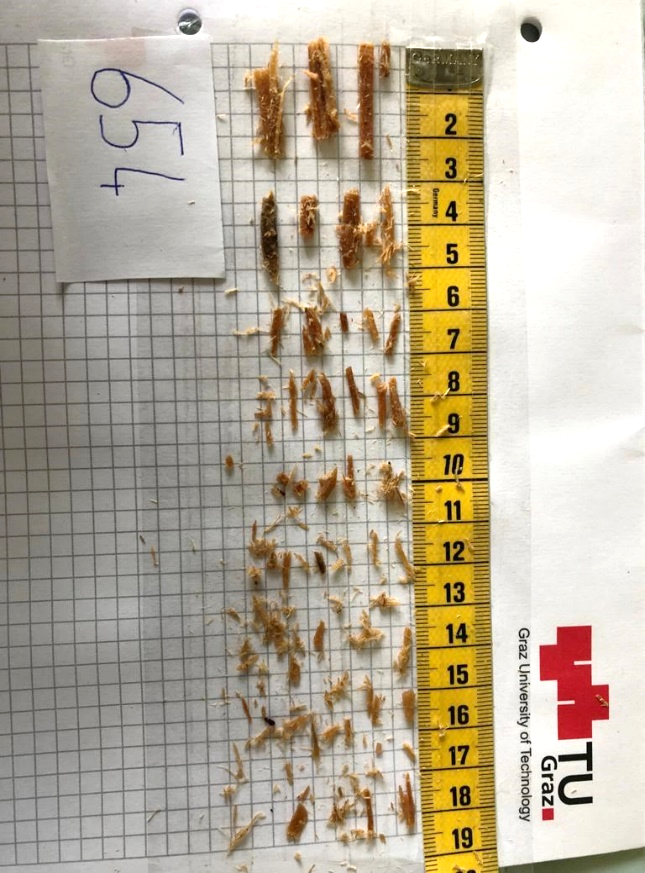


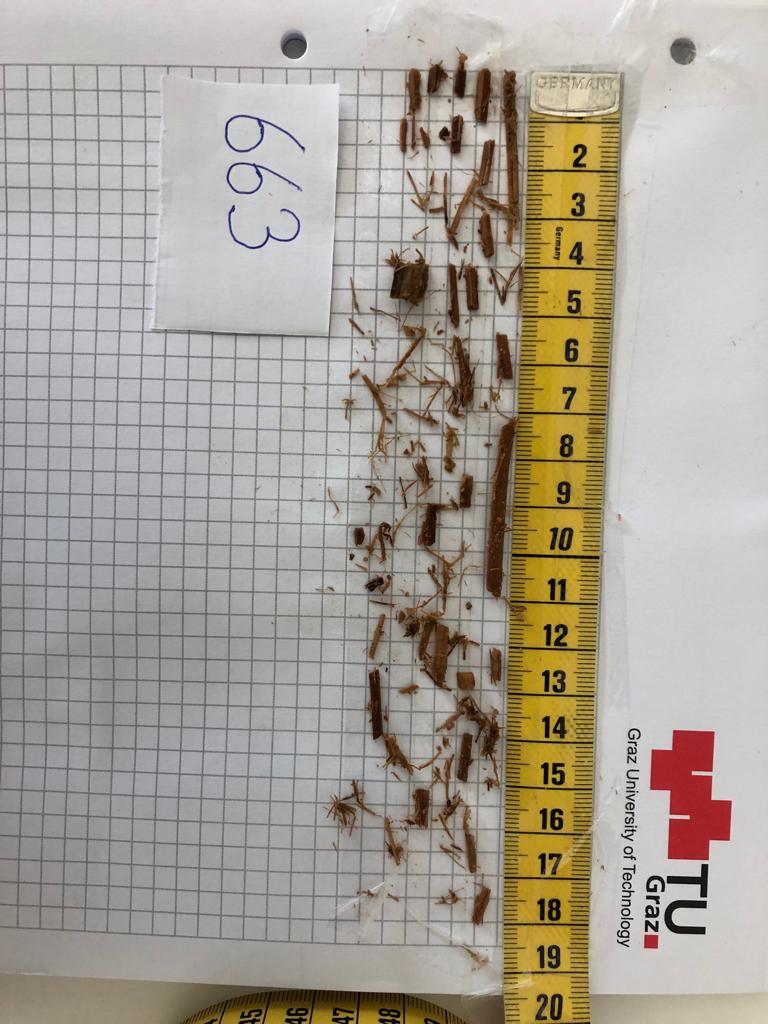

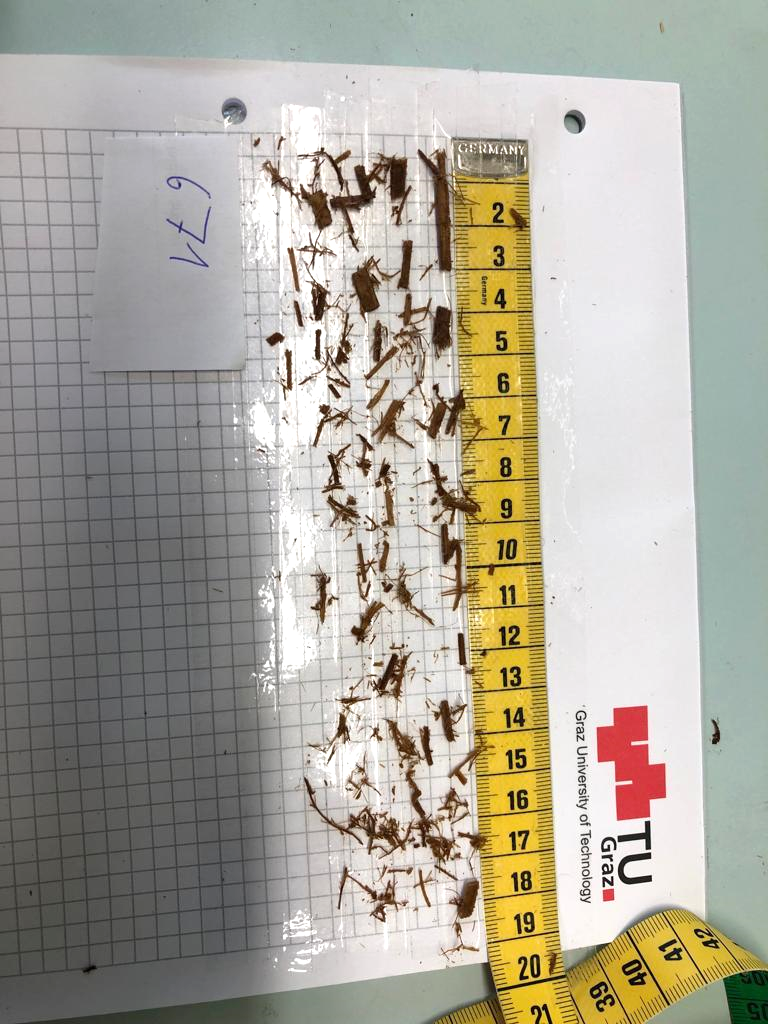

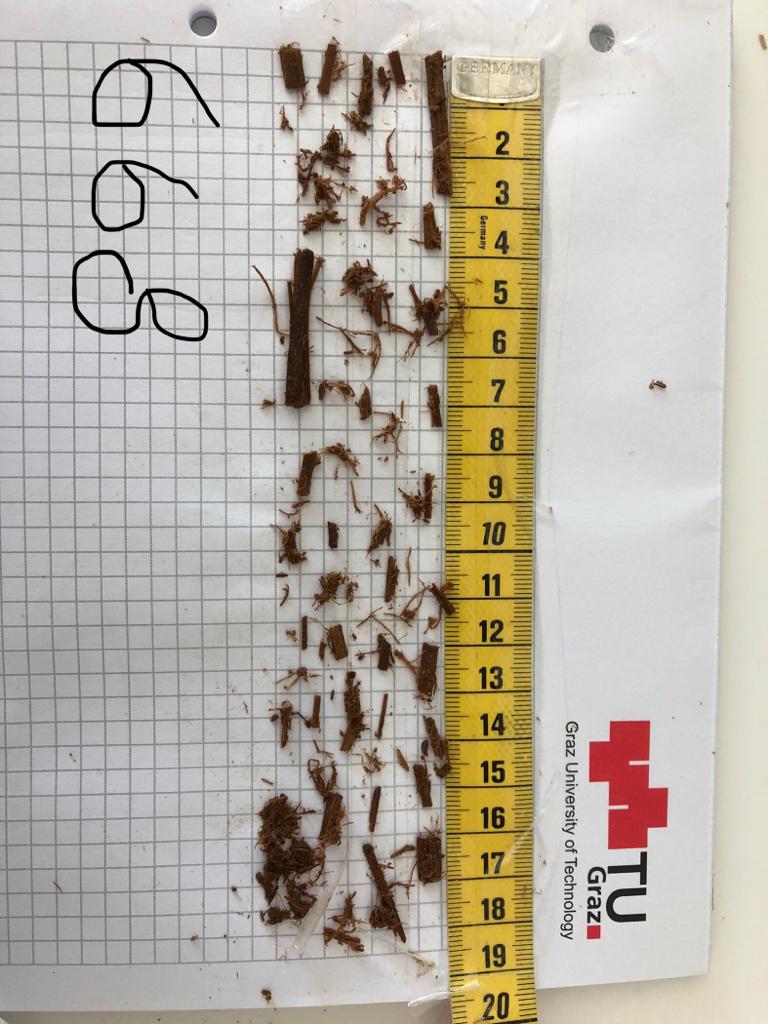

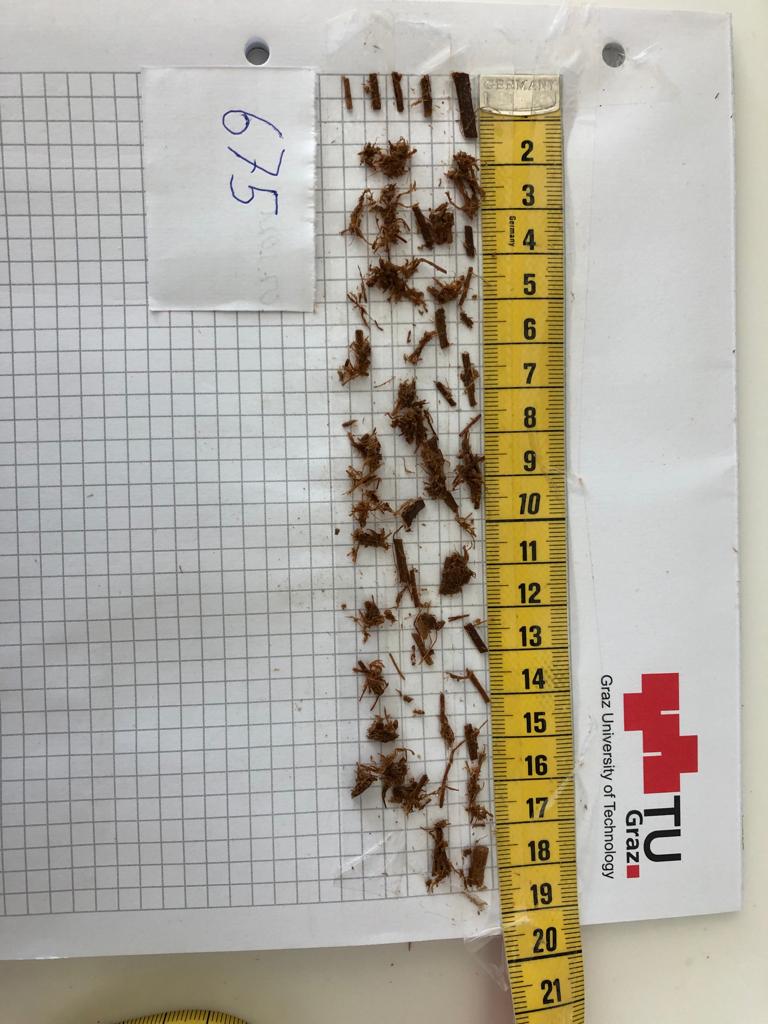
*Figure S2D*. Photos of pretreated pine with scale. 654/D1 steam explosion (190°C/10 minutes); 649/D2 0.5% H_2_SO_4_ + steam explosion (180°C/10 minutes); 657/D3 0.5% H_2_SO_4_ + steam explosion (160°C/10 minutes).

*Figure S2E*. Photos of pretreated miscanthus with scale; 663/E1 0.5% H2SO4 + steam explosion (160°C/10 minutes); 668/E2 0.5% H2SO4 + steam explosion (180°C/10 minutes); 671/E3 steam explosion (180°C/10 minutes); 675/E4 steam explosion (190°C/10 minutes); E: 1% NaOH + steam explosion (180°C/10 minutes).

**3. Hydrolysis and filtration**


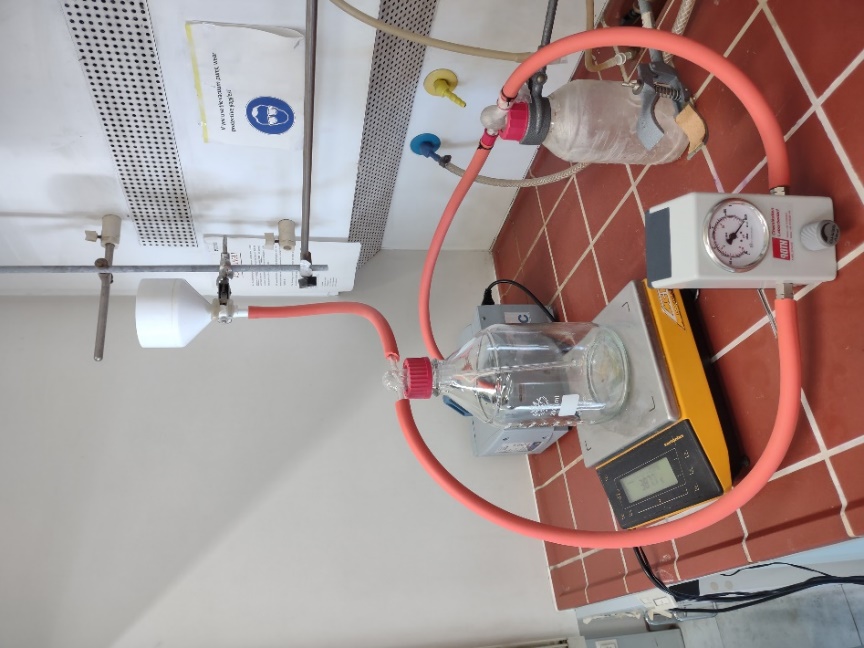


*Figure S3*. Vacuumfiltration set-up. Büchner funnel (PP, 390mL) with two glass fiber filter circles (MN 85/70 BF, 0.6µm from Macherey-Nagel). Vacuum adjusted to 100mbar with vacuum gauge).


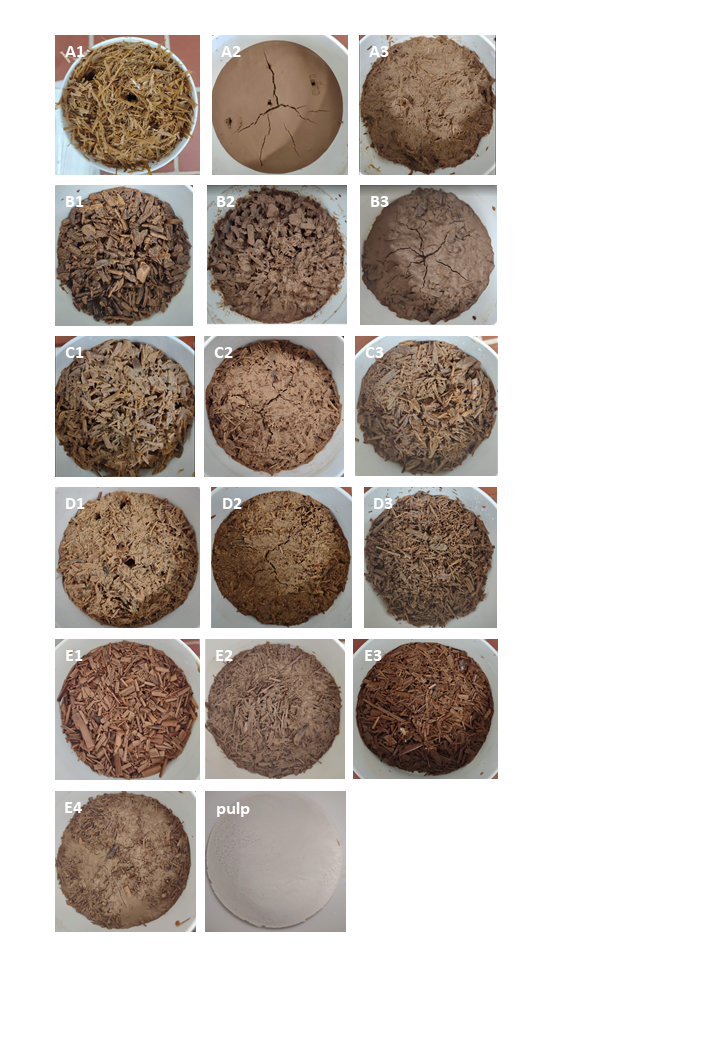


*Figure S4*. Photos of filter cakes obtained by lab-scale vacuum filtration





*Figure S5*. Filtration mass balances including filtrate (dark gray), dried filter cake (gray) and residual filtrate in the wet filter cake (white).

*
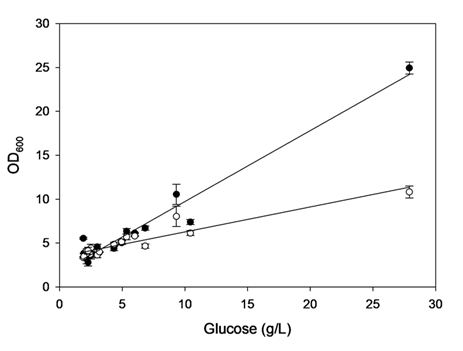
*

*Figure S6*. Correlation of OD600 to glucose concentration in lignocellulose hydrolysates (including pulp sample). Mineral media with 50% lignocellulosic hydrolysates in deep-well plate cultivations of *C. necator* H1G^+^3 (full circles) and *C. neactor* H16 adapted to glucose (open circles).

*
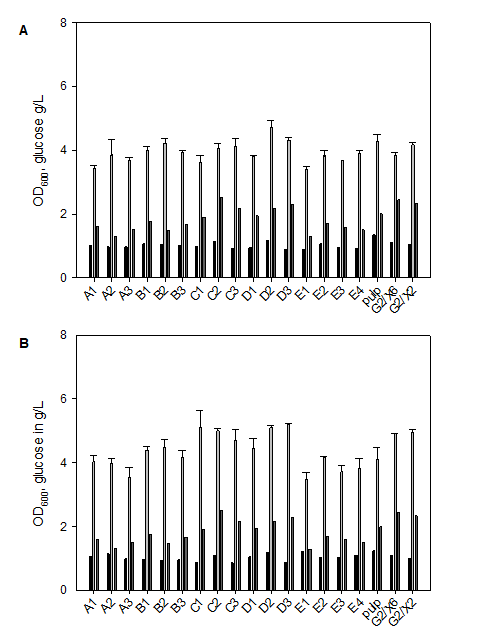
*

*Figure S7*. Growth of *C. necator* strains on different hydrolysates, black bar at 0h (inoculum), light gray bar at after 1 day (26h), dark gray bar glucose concentration in g/L. For comparison, growth in a medium containing 2g/L glucose and 6g/L xylose (G2/X6) or 2g/L xylose (G2/X2) is shown. (A) *C. necator* H16 (DSM 428) adapted to glucose by adaptive laboratory evolution. (B) *C. necator* H1G^+^3 (DSM 545).
